# Observing Without Acting: A Balance of Excitation and Suppression in the Human Corticospinal Pathway?

**DOI:** 10.3389/fnins.2018.00347

**Published:** 2018-05-23

**Authors:** Ricci Hannah, Lorenzo Rocchi, John C. Rothwell

**Affiliations:** University College London Institute of Neurology, London, United Kingdom

**Keywords:** transcranial magnetic stimulation, motor cortex, current direction, mirror neurons, motor resonance

## Abstract

Transcranial magnetic stimulation (TMS) studies of human primary motor cortex (M1) indicate an increase corticospinal excitability during the observation of another's action. This appears to be somewhat at odds with recordings of pyramidal tract neurons in primate M1 showing that there is a balance of increased and decreased activity across the population. TMS is known to recruit a mixed population of cortical neurons, and so one explanation for previous results is that TMS tends to recruit those excitatory output neurons whose activity is increased during action observation. Here we took advantage of the directional sensitivity of TMS to recruit different subsets of M1 neurons and probed whether they responded differentially to action observation in a manner consistent with the balanced change in activity in primates. At the group level we did not observe the expected increase in corticospinal excitability for either TMS current direction during the observation of a precision grip movement. Instead, we observed substantial inter-individual variability ranging from strong facilitation to strong suppression of corticospinal excitability that was similar across both current directions. Thus, we found no evidence of any differential changes in the excitability of distinct M1 neuronal populations during action observation. The most notable change in corticospinal excitability at the group level was a general increase, across muscles and current directions, when participants went from a baseline state outside the task to a baseline state within the actual observation task. We attribute this to arousal- or attention-related processes, which appear to have a similar effect on the different corticospinal pathways targeted by different TMS current directions. Finally, this rather non-specific increase in corticospinal excitability suggests care should be taken when selecting a “baseline” state against which to compare changes during action observation.

## Introduction

A range of evidence illustrates that some neurons in the motor system alter their activity not only during the execution of an action, but also when observing the actions of others (di Pellegrino et al., 1992; Gallese et al., 1996; Kraskov et al., 2009, 2014; Mukamel et al., 2010). These so-called “mirror neurons” were first identified in primate premotor cortex (di Pellegrino et al., 1992; Gallese et al., 1996), where select neurons that responded during the monkey's own grasping movement by increasing their firing rates also increased their firing rates when the monkey observed the same action performed by a human experimenter. Both non-invasive and invasive studies have since pointed to the existence of similar, mirror-like activity in the human motor system, including in the primary motor cortex (M1) (Fadiga et al., 1995; Hari et al., 1998) and supplementary motor area (Mukamel et al., 2010). Transcranial magnetic stimulation (TMS) studies have shown that the corticospinal pathway is facilitated during action observation (Fadiga et al., 1995; Gangitano et al., 2001; Maeda et al., 2002; Labruna et al., 2011; Gueugneau et al., 2015), which seems consistent with the increased activity in primate premotor areas (di Pellegrino et al., 1992; Gallese et al., 1996; Kraskov et al., 2009), with direct cortico-cortical connections to M1, and in M1 itself (Vigneswaran et al., 2013; Kraskov et al., 2014). However, since some of those neurons in M1 included pyramidal tract neurons, the majority of which project directly to the spinal cord (Lemon, 2008), it remains unclear how the increased activity in the motor system during observation is prevented from producing overt movement.

One explanation put forward by Kraskov et al. (2014) is that there is a balance of increased and decreased activity across the population of pyramidal tract neurons that ultimately cancels out so that no movement occurs. This was based on their finding that whilst 29% of M1 pyramidal tract neurons sampled showed increased firing rates during both action and observation, 22% of neurons exhibited increased firing rates during action but suppressed firing rates during observation (Kraskov et al., 2014). These data were consistent with the mix of facilitation and suppression that had previously been observed in neurons in the human supplementary motor area during action observation, which also included neurons that responded oppositely during action and observation (Mukamel et al., 2010). Thus, not only is there a potential balance of facilitated/suppressed activity, which could in itself explain the lack of overt movement, but the structure of activity across the population of neurons can be qualitatively quite different during the observation compared to execution of movement. These different neural states could also explain why no movement is produced (e.g., Kaufman et al., 2014). But if the balanced facilitation/suppression account is correct, why then is the typical finding in TMS studies of an increase in corticospinal excitability when observing an action? Detecting an overall increase in corticospinal excitability might be possible if TMS favored the recruitment of the sub-population of corticospinal output neurons whose activity increased, rather than the sub-population whose activity had decreased.

To test this idea, we took advantage of the directional sensitivity of TMS-evoked corticospinal activity (Day et al., 1989; Di Lazzaro et al., 2001). Epidural recordings indicate that descending corticospinal activity has a lower threshold and shorter onset latency when evoked by posterior-anterior (PA), as opposed to anterior-posterior (AP), induced currents across the central sulcus (Di Lazzaro et al., 2001). The prevailing hypothesis is that PA and AP currents recruit distinct excitatory synaptic inputs to the same corticospinal neurons (Day et al., 1989; Di Lazzaro and Rothwell, 2014). However, it remains a possibility that they each recruit distinct sub-populations of corticospinal neurons, potentially even originating in separate sub-divisions of M1 (e.g., Witham et al., 2016). In a previous study, we showed that motor evoked potentials (MEPs) evoked by PA and AP currents were differentially modulated during the warning period of a reaction time task (Hannah et al., 2018), implying these putative sets of neurons are differentially modulated during action preparation. Here, we predicted that PA- and AP-evoked MEPs would be oppositely modulated during action observation, in a manner akin to mixture of facilitated/suppressed activity of neurons seen in primate studies (Vigneswaran et al., 2013; Kraskov et al., 2014). Specifically, we expected a facilitation of responses to standard PA currents, as is commonly reported (Naish et al., 2014), and an inhibition of responses with AP currents.

## Materials and methods

### Participants

Twenty healthy right-handed volunteers participated (females = 9; mean age: 25 ± 1 years, range 19–32 years) in the study. All provided their written informed consent and reported no contraindications to TMS (Rossi et al., 2011). The experimental protocol was approved by the by University College London Ethics Committee.

### Experimental design

MEPs evoked by TMS applied to M1 were recorded in two intrinsic hand muscles (first dorsal interosseous, FDI; abductor digiti minimi, ADM) whilst participants observed video clips of a hand reaching and using a precision grip (between the index finger and thumb) to pick up and put down a peg. TMS pulses were applied: (i) during an extra-task baseline (B_ET_), where participants observed a blank screen outside the context of the action observation task; (ii) at an intra-task baseline (B_IT_), where participants were engaged in the task but simply observed the blue background in-between video clips; and (iii) during the observation of video clips showing a precision grip. Two blocks of measurements were performed, one with each TMS current direction (PA and AP). The order of current direction was randomized between participants and a 5 min passive rest period separated each block. We were primarily interested in whether MEPs evoked by PA and AP currents were differentially modulated by action observation. To verify that any modulation was a mirror-like effect specific to observation of the precision grip, it was important to show that the effects were time-locked to the observation of the action and that the effect was specific to a muscle predominantly involved in actually performing the movement (Fadiga et al., 1995; Borroni et al., 2005; Naish et al., 2014). To do this we compared responses across time points and muscles, since the FDI is known to be more active during a precision grip than the ADM (Davare et al., 2009). Finally, MEP onset latencies were recorded for PA and AP currents at the end of the experiment as a proxy for the latency of corticospinal activity, which allows us to confirm that the two current directions recruit distinct populations of neurons (Day et al., 1989; Di Lazzaro et al., 2001; Hamada et al., 2013).

### Action observation stimuli

**Figure 1:**
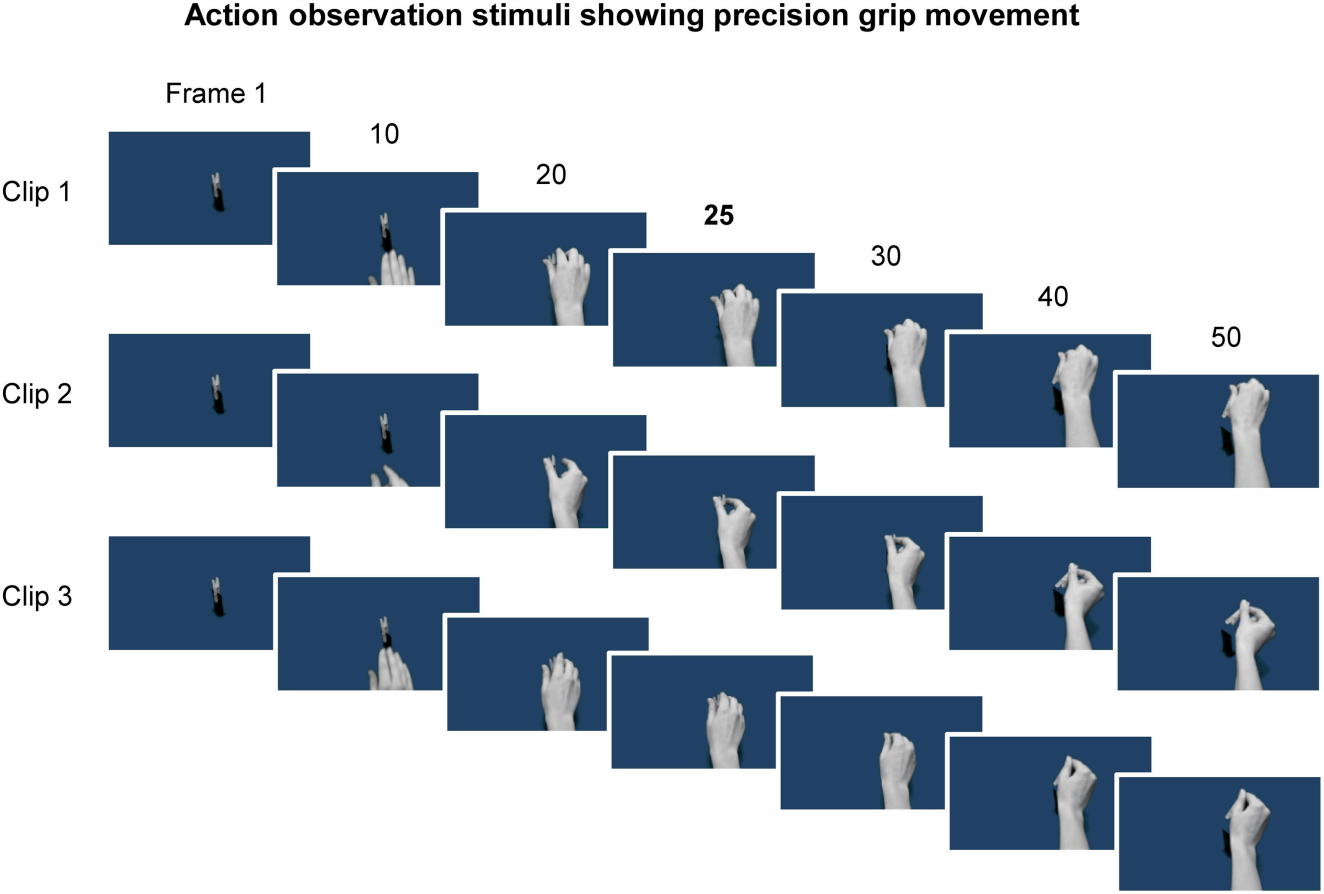
Example frames from the three video clips showing a hand reaching and using a precision grip to pick up a peg. Representative frames from the first half of each clip, which consisted of 100 frames in total, are shown. The 25th frame is shown as this frame reflected the timing of contact between the hand and the object, and was also the time at which the TMS pulse was delivered. Note that the kinematics are slightly different for each of the three clips.

Three video clips were presented. Each consisted of the same hand performing a precision grip to manipulate the same object on a different occasion (Figure 1). The video clips were matched for movement duration, by initially selecting from a larger pool of clips, and edited (via Motion 2 software as part of the Final Cut Studio application package) to consist of video 100 frames each with a consistent point of contact between hand and object at the 25th video frame. Each frame was presented for ~33 ms, i.e., two computer screen refreshes at a rate of 60 Hz, such that video clips lasted 3.3 s. Since movement duration was approximately the same, the main difference between the video clips was in the kinematics, for example small differences in grip shaping. The model in the videos was female and all actions were filmed from the egocentric perspective, since there is some evidence that observing actions from the first person point of view yields the largest enhancement of MEPs (Alaerts et al., 2009).

### Surface electromyogram (EMG)

EMG activity was recorded from the right FDI and ADM muscles. Electrodes were placed in a belly-tendon arrangement over the muscles. The ground electrode was over the styloid process of the radius. Signals were amplified with a gain of 1000 (Digitimer, UK), band-pass filtered (5–3,000 Hz), digitized at 5 kHz (Power1401; CED, Cambridge, UK), and analyzed with Signal v5.10 software (Cambridge Electronic Design, UK). EMG recordings enabled measurement of MEPs and the detection of any volitional muscle activity during the task.

### Transcranial magnetic stimulation (TMS)

The FDI representation of the left primary motor cortex was stimulated via a TMS device connected to a figure-of-eight coil (Magstim 200^2^, The Magstim Co. Ltd., UK). The coil was held tangentially on the scalp at an angle of 45° to the mid-sagittal plane to induce a posterior-anterior (PA) current across the central sulcus (Sakai et al., 1997). The motor hot spot was found by searching for the position where slightly suprathreshold PA currents produced the largest and most consistent MEPs in FDI at rest. The position was marked on a cap worn by the participants. The coil was held to induce either a PA current across the central sulcus, or an oppositely directed AP current, whereby the position of the coil handle was reversed around the intersection of coil windings (Sakai et al., 1997). The inter-pulse interval for TMS stimuli outside of the action observation task was 5 ± 0.5 s, and during the task it was ~7 s.

Resting motor threshold (RMT) was defined as the lowest intensity to evoke an MEP in the FDI of at least 0.05 mV in five of 10 consecutive trials while subjects were at rest. The test stimulus intensity during the task was set low to elicit a small MEP (~0.5 mV) in the FDI and facilitate the selective recruitment of different neuronal populations responsible for early and late corticospinal activity. At high stimulus intensities the potential to recruit distinct neuronal populations with different current directions is diminished because pulses tend recruit a mixed population (Di Lazzaro et al., 2001). Despite the hotspot not being optimized for eliciting MEPs in the ADM, the hotspot and intensity were still generally sufficient to evoke small MEPs in the ADM.

MEP onset latencies were determined for the FDI during weak background muscle activity, again to ensure that low stimulus intensities could be used thereby maximizing the likelihood of selectively recruiting early or late arriving MEPs with PA or AP currents (Day et al., 1989; Hamada et al., 2013; Hannah and Rothwell, 2017). Active motor threshold (AMT) was defined as the lowest intensity to evoke a discernible MEP in the FDI in five of 10 consecutive trials while subjects maintained a voluntary isometric finger abduction sufficient to produce 5–10% of maximum voluntary EMG amplitude, and was measured with PA and AP currents. Thereafter, 20 MEPs were measured for each current direction during isometric finger abduction at 5–10% maximum EMG amplitude and with a stimulus intensity equal to 110% AMT.

### Experimental procedures

**Figure 2:**
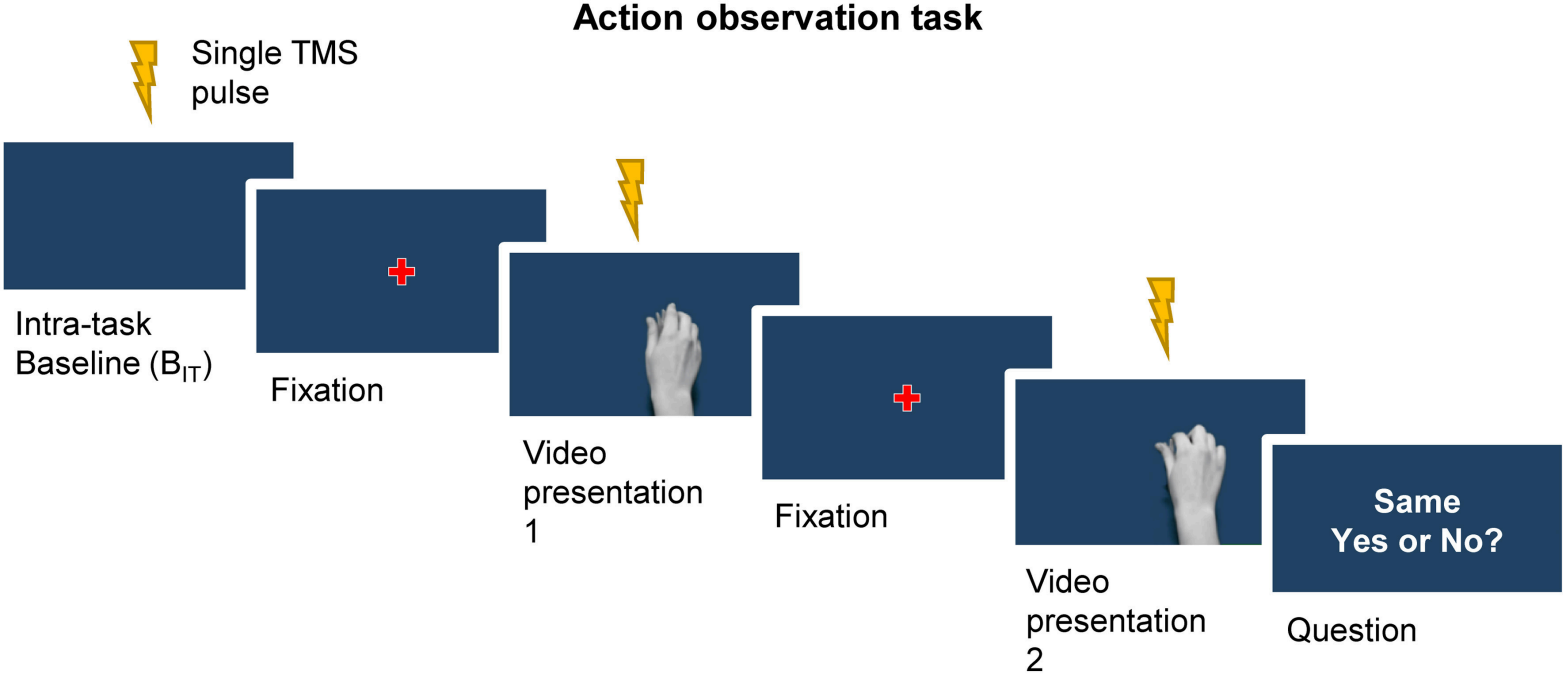
A single trial of the action observation task began with a TMS pulse being delivered whilst participants focused their gaze on the blue background of the screen (intra-task baseline, B_IT_). This was followed by the appearance fixation cross, and in turn the first presentation of a video clip. A second fixation cross preceded the second presentation of a video clip. The trial ended with a question presented on the screen regarding the sameness of the two videos, to which participants verbally responded. Single TMS pulses were delivered during the presentation of the intra-task baseline period, as well as at the point of contact with the object during each of the two video presentations.

Participants were seated comfortably in a dimly lit room, ~60 cm in front of a computer screen with their hands resting on a pillow positioned on their lap, underneath a desk and out of view. After a brief familiarization with the task and recording of the extra-task baseline MEPs (20 MEPs in total) for a given current direction, participants performed the first block of the action observation task (Figure 2). The task which consisted of 24 trials, with TMS pulses being delivered at three separate times during each trial. Trials started with a single TMS pulse being delivered whilst participants focused on the blue background presented on the screen (intra-task baseline). Following a 5 s delay, a red fixation cross was presented for 1 s at the center of screen and participants were instructed to fix their gaze on it. This was followed by the first presentation of a video clip, during which a single TMS pulse was delivered. After a short delay (3 s) another fixation cross was then presented for 1 s prior to commencing the second presentation of a video clip, where another single TMS pulses was delivered. Within each trial the video presentation could consist of either the same video clip presented twice (50% of trials) or two different video clips being presented once each (50% of trials). Each clip was shown the same number of times in total and the number of times each clip appeared first or second in a trial was also balanced. Participants were instructed to carefully observe the grip shaping during object manipulation, and were asked after the second video in a pair to verbally respond to a question presented on the screen (2 s duration) asking whether the two video clips in a trial were the same or different. This served two purposes: (1) to ensure participants paid particular attention to the kinematic features of the grip; and (2) to maintain their vigilance during the task. Responses were recorded throughout the experiment for further analysis. The next trial began after a further 3 s delay, during which the blue background was presented. This completed the trial. Each block lasted ~8 min, including a short break of 1 min midway through the block to allow participants to rest.

During the task participants were encouraged to keep their arms and hands still and their muscles relaxed, and received verbal feedback throughout on the presence of voluntary EMG activity from the FDI and ADM in order to minimize the presence of volitional muscle activity. TMS pulses were delivered at the time of contact with the object in each video clip (i.e. 25th frame) because primate studies have shown that the population activity of corticospinal mirror neurons is modulated most strongly at or just before the time of object displacement, i.e., very close to the time of object contact (Vigneswaran et al., 2013; Kraskov et al., 2014). The task, including the presentation of video clips and control of TMS stimuli, was programmed in MATLAB R2013b (MathWorks, Natick, USA) with the Cogent toolbox (UCL LON, UK) used to manage the presentation of the videos.

### Data analysis

MEP peak-to-peak amplitude was measured on a trial-by-trial basis, and averaged across all trials for each time point (extra-task baseline, intra-task baseline, video presentation 1 and 2) and current direction (PA and AP). To ensure that MEP amplitudes were not contaminated by volitional muscle activity, the peak-to-peak EMG amplitude was measured in the 100 ms prior to the TMS pulse. Trials were included for analysis if the peak-to-peak EMG amplitude in the prior 100 ms was < 0.05 mV. This resulted in the exclusion of 6 ± 1 % of trials (equivalent to 4 out of 72 MEPs per block) being removed. The proportion of correct responses to the discrimination aspect of the task was calculated for PA and AP blocks separately and expressed as a percentage. The mean onset latency of MEPs measured during active muscle contraction was determined visually from the raw EMG traces for each current direction separately (Day et al., 1989; Hamada et al., 2013).

### Statistical analysis

Paired *t*-tests were used to compare motor thresholds (RMT, AMT), test stimulus intensities and MEP onset latencies across PA and AP current directions. One sample *t*-tests were used to examine whether response accuracy on the discrimination aspect of the task was greater than chance for PA and AP blocks.

Data are reported as group mean ± standard error of the mean (SEM). Repeated measures ANOVA (rmANOVA) was used to evaluate the majority of the data. *P-*values < 0.05 were considered significant. Where necessary, the Greenhouse-Geisser procedure was applied to correct for violations of sphericity in ANOVA. First, a two-way rmANOVA was used to determine the effects of current direction (PA, AP) and muscle (FDI, ADM) on extra-task baseline MEP amplitudes in order to confirm similar amplitude of MEPs for PA and AP currents at baseline. We were then interested in evaluating whether there were any changes in corticospinal excitability from the extra- to the intra-task baseline, as this could reveal potential changes that were not temporally linked to the observation of the precision grip, and which might therefore influence our interpretation of any changes over time. Two-way rmANOVA was used to evaluate the effects of baseline time point (B_ET_, B_IT_), current direction (PA, AP) and muscle (FDI, ADM) on absolute MEP amplitudes. Having established that MEPs in the FDI and ADM were significantly increased compared to the extra-task baseline simply by virtue of being engaged in the task (see results), indicating effects that were not time-locked to observation of the action, we decided to exclude data at the extra-task baseline from further analysis.

Since we expected no explicit differences in MEP amplitudes between the first and second presentation of videos during a trial, we averaged across the two presentation time points for each current direction and muscle to create a single variable named “precision grip (PG).” Thus, to evaluate the influence of action observation on PA and AP evoked MEPs, rmANOVA was used to test the effect of time point (B_IT_, PG), current direction (PA, AP) and muscle (FDI, ADM) on absolute MEP amplitudes.

As a further test of any muscle-specific changes in MEP amplitudes, we calculated the ratio of FDI:ADM MEP amplitudes on an individual basis for the extra- and intra-task baseline, and for the precision grip. Two-way ANOVA was performed to evaluate the changes across baselines [i.e., effect of current direction (PA, AP) and time point (B_ET_, B_IT_)] and across time points within the task [i.e., effect of current direction (PA, AP) and time point (B_IT_, PG)].

For each current direction, MEP amplitudes of the FDI were normalized to the respective values at the extra-task and intra-task baseline by expressing them as a ratio (e.g., precision grip/intra-task baseline), such that values >1 indicate a facilitation of MEPs during observation of the precision grip compared to the intra-task baseline, and values <1 indicate a relative suppression. Normalized data are shown for illustrative purposes.

Since we did not observe the prototypical facilitation of MEPs in response to observing the precision grip (see results), we performed a *post hoc* exploratory analysis of individual differences in order to try and characterize individual responsiveness to the task. To do this the percentage of individuals demonstrating facilitation and inhibition of MEPs relative to the intra-task baseline were calculated. Finally, Pearson's correlation was used to evaluate the relationship between AP and PA MEP amplitudes normalized to the intra-task baseline.

## Results

### Motor thresholds and MEP latencies

RMT [*t*_(19)_ = −8.81, *P* ≤ 0.001], AMT [*t*_(19)_ = −8.41, *P* ≤ 0.001) and the 0.5 mV test stimulus intensity [*t*_(19)_ = −7.40, *P* ≤ 0.001] were all significantly greater for AP compared to PA currents (Table 1). MEP latencies were greater for AP compared to PA currents by ~2 ms on average [*t*_(19)_ = −11.547, *P* ≤ 0.001]. These data are all consistent with previous reports (Day et al., 1989; Sakai et al., 1997; Hamada et al., 2013). Despite this difference in latencies, MEP amplitudes were similar for both current directions during the extra-task baseline. ANOVA revealed no main effect of current direction [*F*_(1, 19)_ = 0.173, *P* = 0.683] or muscle × current direction interaction [*F*_(1, 19)_ = 0.153, *P* = 0.700], but there was a significant main effect of muscle which indicated that MEP amplitudes were greater for the FDI compared to ADM muscle [*F*_(1, 19)_ = 17.920, *P* < 0.001]. This was to be expected given that the motor hotspot used was based on the optimal TMS coil position and orientation for FDI, not the ADM.

**Table 1:** Motor thresholds and MEP latencies for each TMS current direction.

**Current direction**	**RMT (%MSO)**	**AMT (%MSO)**	**0.5 mV intensity (%MSO)**	**MEP latency (ms)**
PA	43 ± 2	33 ± 1	50 ± 2	22.3 ± 0.4
AP	54 ± 2	44 ± 2	61 ± 2	24.4 ± 0.4

### Discrimination performance during the action observation task

Participants correctly answered the question regarding the sameness of the video clips on 87 ± 2% and 86 ± 2% of trials respectively for PA and AP blocks, with performance levels being greater than chance in each case [*t*_(19)_ = 23.294, *P* ≤ 0.001 and *t*_(19)_ = 17.8, *P* ≤ 0.001].

### Extra- vs. intra-task baseline

**Figure 3:**
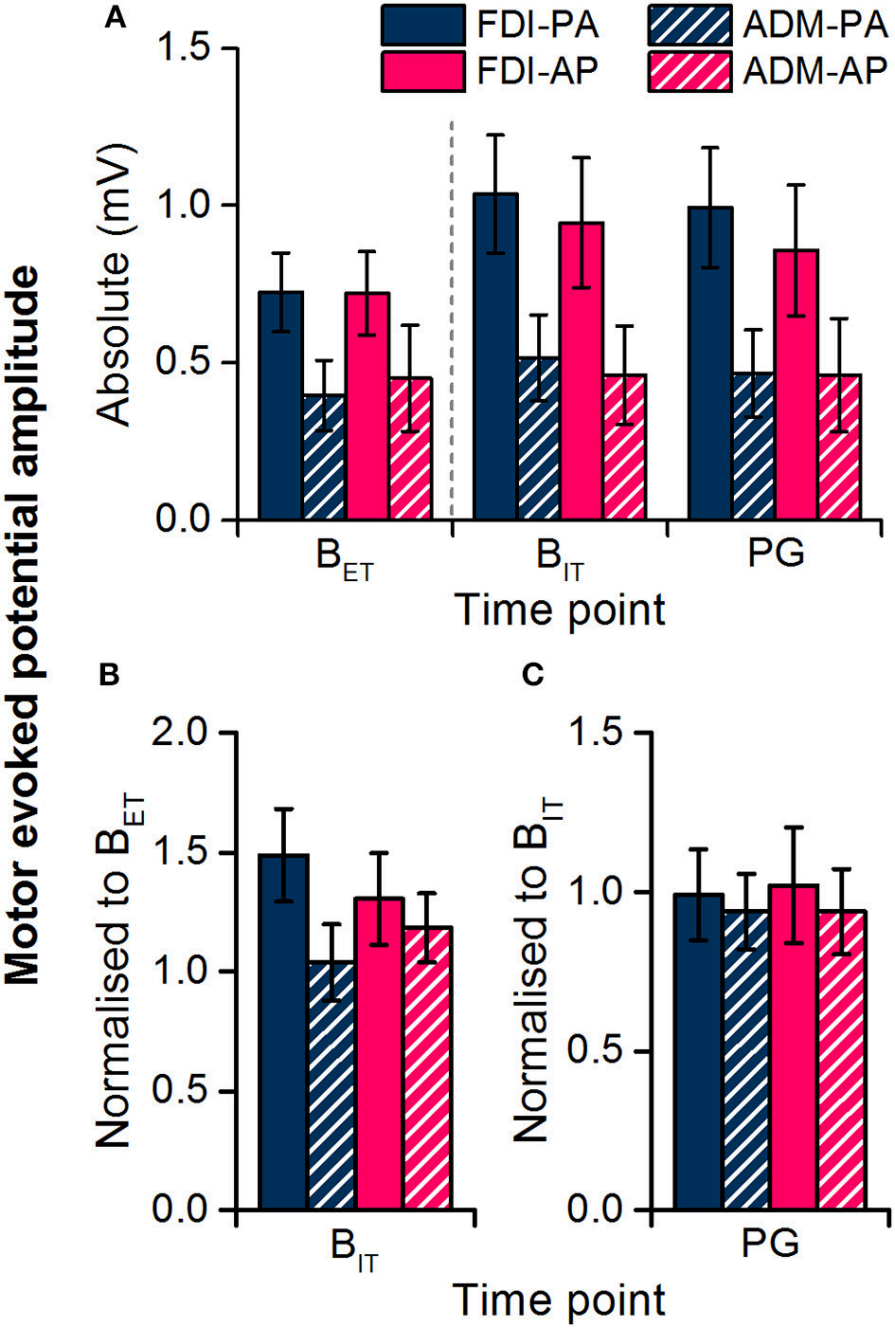
Motor evoked potential amplitudes recorded from the FDI and ADM muscles during the extra-task baseline (B_ET_) and the action observation task (intra-task baseline, B_IT_; precision grip, PG). Dashed line separates measurements made outside of the observation task (B_ET_) from those within the task (B_IT_, PG). Data are shown in absolute terms **(A)** and normalized to extra-task baseline **(B)** or intra-task baseline **(C)**.

We first compared MEP amplitudes at the extra-task baseline, measured prior to the observation task, with those at the intra-task baseline, measured during the task. The data showed a significant main effect of baseline time point (Figures 3A,B, Table 2), which indicated that MEP amplitudes were greater at the intra- compared to the extra- task baseline, irrespective of current direction and muscle. There was also a main effect of muscle (Table 1), which indicated that MEP amplitudes on the whole were greater for the FDI than the ADM (*P* < 0.001).

**Table 2:** ANOVA on absolute MEP amplitudes comparing muscles (FDI, ADM), current directions (PA, AP) and time point (B_ET_, B_IT_).

**Effect**	***F*-ratio**	***P***
Current direction	*F*_(1, 19)_ = 0.129	0.724
Muscle	*F*_(1, 19)_ = 29.428	<0.001
Time point	*F*_(1, 19)_ = 5.909	0.025
Current direction × muscle	*F*_(1, 19)_ = 2.312	0.145
Current direction × time point	*F*_(1, 19)_ = 1.798	0.196
Muscle x time point	*F*_(1, 19)_ = 3.355	0.083
Current direction × muscle × time point	*F*_(1, 19)_ = 0.014	0.907

**Figure 4:**
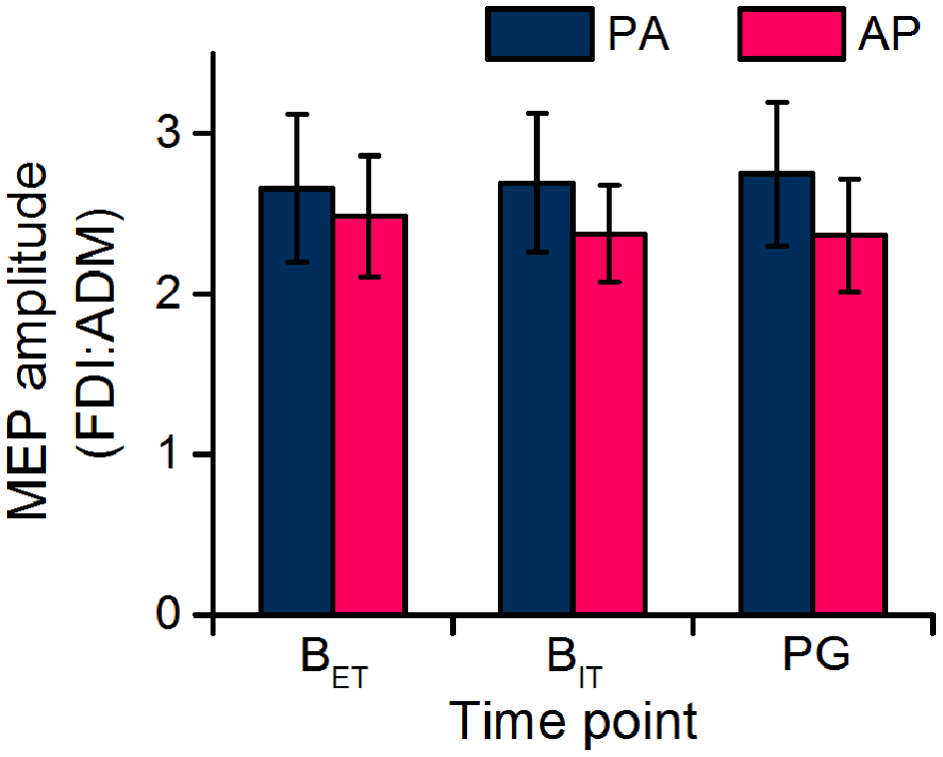
Ratio of FDI and ADM MEP amplitudes recorded during the extra-task baseline (B_ET_) and the action observation task (intra-task baseline, B_IT_; precision grip, PG).

The FDI:ADM MEP ratio remained constant over time (Figure 4) confirming that the modulation of MEP amplitudes across time points was not specific to the FDI. In the statistics the ANOVA showed no main effect of current direction [*F*_(1, 19)_ = 1.258, *P* = 0.276] or time point [*F*_(1, 19)_ = 0.016, *P* = 0.900], or any interaction [*F*_(1, 19)_ = 0.123, *P* = 0.729].

### Intra-task baseline vs. precision grip observation

In light of the seemingly non-specific increase in MEP size at the intra-task baseline, i.e., the increase was not time-locked to observing the action nor limited to the movement-relevant FDI muscle, we excluded the extra-task baseline MEP measurements from subsequent analysis and used the intra-task baseline MEPs as a reference for comparing MEPs during the observation of the precision grip. In this way, any changes ought to be directly due to observation of the action. Overall, there was no effect of action observation on MEPs for either current direction or muscle (Figures 3A,C), as indicated by a lack of a main effect or interaction with time point in the rmANOVA (Table 3). The only significant result was a main effect of muscle indicating that MEPs were greater for the FDI than ADM overall.

**Table 3:** ANOVA on absolute MEP amplitudes comparing muscles (FDI, ADM), current directions (PA, AP) and time point (B_IT_, PG).

**Effect**	***F*-ratio**	***P***
Current direction	*F*_(1, 19)_ = 0.610	0.444
Muscle	*F*_(1, 19)_ = 20.139	<0.001
Time point	*F*_(1, 19)_ = 0.610	0.444
Current direction x muscle	*F*_(1, 19)_ = 2.093	0.164
Current direction x time point	*F*_(1, 19)_ = 0.000	0.987
Muscle x time point	*F*_(1, 19)_ = 0.238	0.632
Current direction x muscle x time point	*F*_(1, 19)_ = 1.199	0.287

The FDI:ADM MEP ratio again remained constant over time (Figure 4) confirming that there were no muscle-dependent changes in MEPs across time points. In the statistics the ANOVA showed no main effect of current direction [*F*_(1, 19)_ = 1.914, *P* = 0.183] or time point [*F*_(1, 19)_ = 0.007, *P* = 0.932], or any interaction [*F*_(1, 19)_ = 0.047, *P* = 0.832].

### Inter-individual variability of responses to action observation

**Figure 5:**
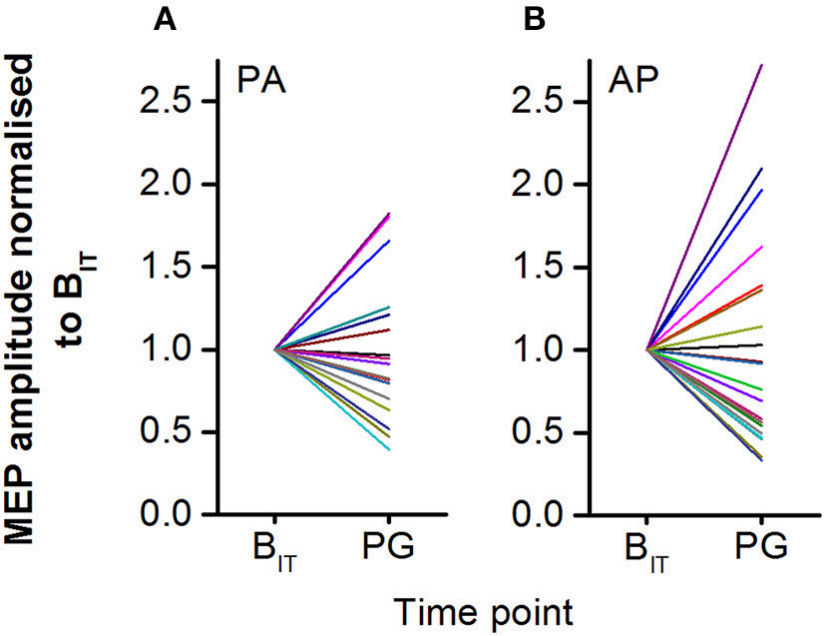
Individual MEP amplitudes for PA **(A)** and AP **(B)** currents shown normalized to the intra-task baseline. MEP responses to action observation during the videos were highly variable across participants, with some showing strong facilitation and others showing strong inhibition.

Large inter-individual differences in response to observing the precision grip were found for both PA and AP TMS currents (Figures 5A,B). For PA currents, 35% of individuals showed a facilitatory effect of observing the precision grip whilst 65% showed an inhibitory response. For AP currents the results were broadly similar, with 40% of individuals showing facilitation and 60% showing inhibition. Normalized PA and AP MEP amplitudes were moderately correlated with each other (*r* = 0.73, *P* < 0.001).

## Discussion

The current experiment attempted to exploit the directional sensitivity of corticospinal output to TMS in order to test the hypothesis that different sub-populations of neurons in the motor cortex responded differentially to action observation. The results showed that there was no difference in the response of MEPs evoked by PA and AP currents at any point during the experiment, and both sets of responses were actually closely related when observing the precision grip movement. In fact, at the group level the main finding was of an increase in corticospinal excitability during the action observation task that was not time-locked to the observation of the movement or specific to the muscle predominantly involved in performing the movement. Thus, the change in corticospinal excitability was not a true mirror-like response. Instead the results point to a top-down modulation of corticospinal excitability, for example by attention- or arousal-related processes, rather than by action observation *per se*. The lack of a true mirror-like effect meant that we were unable to confirm or reject our hypothesis. However, the similar increase of excitability exhibited by PA- and AP-evoked MEPs during the task might imply that cognitive states such as arousal have a similar effect on PA- and AP-sensitive neurons.

### Temporally non-specific increase in corticospinal excitability

A recent review by Naish et al. (2014) suggested that the most common finding in TMS studies of action observation was an increase in corticospinal excitability compared to some baseline measure taken inside or outside the context of the task. As a first step toward checking that any increase was specific to observing the precision grip in our videos, we first compared MEPs at the extra- and intra-task baseline. Here simply observing the blue background screen within the task resulted in a facilitation of corticospinal excitability compared to the extra-task baseline where participants were not engaged in the action observation task and simply observed a black screen. Thus, this increase was temporally unrelated to the observation of the precision grip.

Since there was no difference in corticospinal excitability between the intra-task baseline and precision grip time points, one could argue that the rise in excitability during the task compared to the extra-task baseline was due to a non-time locked effect of action observation, i.e., a carry-over effect lasting beyond the observation period and into the intra-task baseline (Labruna et al., 2011). This seems unlikely, however, as recordings of mirror neurons in primate (Vigneswaran et al., 2013; Kraskov et al., 2014) and human motor areas (Mukamel et al., 2010) indicate a phasic, rather than sustained, modulation of the population activity that returns to baseline levels within a few seconds, coincident with the completion of the observed action/cessation of the stimulus. Incidentally, this phasic modulation has a broadly similar time course to that seen when actually performing the action. Furthermore, whilst evidence in TMS studies of humans for a close temporal correspondence between observed actions and changes in corticospinal excitability is mixed (Naish et al., 2014), Borroni and colleagues have repeatedly demonstrated a clear phasic modulation of corticospinal and spinal excitability during the observation of cyclic wrist movements (Borroni et al., 2005; Puglisi et al., 2017). Together these studies would seem to argue against the notion of carry-over effects being important here.

An alternative explanation for the lack of a change in corticospinal excitability when observing the precision grip could be that the timing of the TMS pulse, at the point of object contact, was sub-optimal. For example, there is evidence that corticospinal excitability rises to a peak a few hundred milliseconds prior to object contact and then begins to return to baseline (Gangitano et al., 2001). Others have also indicated a decrease in excitability toward the point of object contact (Lago and Fernandez-del-Olmo, 2011). If this were true, our TMS stimulus may have been too late to detect any potential facilitation of corticospinal excitability. However, the movements in those studies were performed very slowly, such that object contact occurred 3.5–5.4 s after video onset. In those cases it is possible that any useful visual information had already been extracted prior to the point of contact and this could explain the relative decrease in excitability thereafter. However, we would argue that the timing of the TMS pulse in our task was well-placed to detect any change in excitability. First, precision grip movements in our videos were performed more quickly, with the hand only coming into view ~200 ms from video onset and object contact occurring ~500 ms later, so that there was much less time available to extract relevant information. Second, the discrimination element of our task required participants to continue to attend to the hand after the TMS pulse because the video clips shared broadly similar kinematics until shortly after that point. The high level of accuracy in the discrimination aspect of the task implies that participants carefully attended to the kinematic cues in each of the video clips. Finally, the population activity of corticospinal mirror-neurons in primate M1 is modulated such that it builds-up to a peak at or just before object displacement (i.e., close to the time of object contact; Vigneswaran et al., 2013; Kraskov et al., 2014). We are confident therefore that the timing of our TMS stimulus was appropriate, though we acknowledge that the use of only one time point may have limited our ability to detect potential changes at other times during the movement.

Issues concerning non-specific effects associated with “baseline” measurements have been discussed previously (Naish et al., 2014). In line with those considerations the data here urge caution against directly comparing corticospinal excitability during action observation to a baseline measured outside the context of the task in order to avoid incorrectly attributing any changes to mirror-like activity.

### Lack of muscle-specific modulation of corticospinal excitability

An alternative criteria for confirming the presence of mirror-like effects is that any modulation of corticospinal excitability should be relatively focal and preferentially target muscles that would be involved in performing the action being observed (Fadiga et al., 1995; Borroni et al., 2005; Naish et al., 2014). However, in the present study we found no evidence of muscle specific changes in MEP amplitudes. The increase in MEP amplitudes during the task was not specific to the FDI muscle [an agonist during precision grip (Davare et al., 2009)] compared to the ADM [minimally active during precision grip (Davare et al., 2009)]. In particular there was no evidence of a muscle-specific modulation of MEPs during the precision grip observation compared to the intra-task baseline. Whilst numerous other studies have established muscle-specific effects during action observation (e.g., Alaerts et al., 2009; Gueugneau et al., 2015; Bunday et al., 2016; Hilt et al., 2017; see also Naish et al., 2014 for a review), we note that sometimes these results stem from muscle by movement interactions (i.e., comparisons across movement conditions) that do not consider whether there has actually been a change relative to some other time point or baseline (Alaerts et al., 2009; Bunday et al., 2016).

### Cognitive state modulates corticospinal excitability

The present findings are consistent with a modulation of corticospinal excitability by cognitive states such as attention or arousal (Gandevia and Rothwell, 1987; Ruge et al., 2014), whereby attending to a muscle/the skin overlying a muscle or to a visual search task can strongly influence the corticospinal output pathway even when it does not involve observing or making any movement. Since the effects of attending to a specific area of one's body can produce quite focal (i.e., muscle-specific) effects (Gandevia and Rothwell, 1987; Ruge et al., 2014), one explanation for the non-specific effects in our study could be that elevated arousal lead to a general increase in corticospinal excitability.

Previous research has highlighted the fact that states of arousal or attention can interact with action observation effects on the motor system (Naish et al., 2014; Betti et al., 2017; Puglisi et al., 2017; Wright et al., 2018). For example, attending to the object to be interacted with can produce a stronger effect on corticospinal excitability than freely observing the scene, whilst the effect when attending specifically to the digits involved in the movement fell somewhere in between (Wright et al., 2018). On the other hand, attending to another task (e.g., counting flashes of light) whilst implicitly observing a movement reduces the size of the change (Puglisi et al., 2017). Given the heterogeneous responses seen at the individual level in our study (Figure 5), one might speculate that individual differences in the locus of attention or attentional resources consumed by the discrimination task contributed to the individual responsiveness to observing the precision grip. In our task, participants were instructed attend to the grip shape and asked to discriminate between video clips in each trial. The former would be expected to confer a moderate, though perhaps still sub-optimal, benefit over no instructions and thus seems unlikely to explain the lack of corticospinal modulation during the task. The latter could conceivably have reduced, but should not have abolished, any mirror-like effect, as many other studies have also included attentional components to maintain vigilance. Finally, participants were explicitly told keep their hands still and relaxed prior to each block of the task, and verbally reminded during the task to relax their hands when volitional activity was detected in either muscle. It is possible that in an attempt to suppress any unwanted volitional movements participants also suppressed possible mirror-like activity in the motor system, and this could then explain the lack of corticospinal facilitation.

### Neurophysiology of changes in the corticospinal output pathway

MEP onset latencies were longer for AP currents compared to PA currents by ~2 ms, consistent with the idea that they preferentially recruit at least partly dissociable populations of neurons in M1 (Day et al., 1989; Hanajima et al., 1998; Di Lazzaro and Rothwell, 2014; Hannah and Rothwell, 2017). However, the increase in corticospinal excitability during the action observation task was similar for both PA and AP TMS currents. Different TMS current directions could in theory activate distinct populations of corticospinal neurons or distinct populations of excitatory synaptic inputs to the same corticospinal neurons (Day et al., 1989; Di Lazzaro and Rothwell, 2014). The present results could therefore be interpreted in two ways. One is that attention- or arousal-related processes have a similar effect on the distinct populations of corticospinal neurons targeted by PA and AP currents. The other is that those processes do not exert their effect via a particular excitatory input pathway to the corticospinal output neurons, but instead target the corticospinal neurons such that input from either pathway is facilitated. On the other hand, as we did not find a specific effect of action observation on corticospinal excitability we were unable to address our original hypothesis concerning the possible pathways involved and direction of changes. However, the correlation between normalized PA and AP responses when observing the precision grip might tentatively be taken to suggest that they are modulated during action observation in a similar manner.

## Conclusions

In conclusion, the present study found no evidence of temporal- or muscle-specific changes in corticospinal excitability that would indicate mirror-like activity. We were therefore unable to address our original question regarding the nature of corticospinal activity during action observation. Instead the presence of a non-specific increase in corticospinal activity common to both TMS current directions might indicate that attention or arousal facilitate the corticospinal output pathway directly rather than through a specific input pathway. Finally, the results again confirm that comparison to an extra-task baseline is not sufficient to establish specific effects of action observation on corticospinal excitability.

## Author contributions

RH and JR contributed to the design of the work. RH acquired and analyzed the data. RH JR, and LR contributed to the interpretation of data. RH drafted the manuscript. RH, JR, and LR contributed to manuscript revision and all read and approved the submitted version.

### Conflict of interest statement

The authors declare that the research was conducted in the absence of any commercial or financial relationships that could be construed as a potential conflict of interest.
